# Constitution and By-Laws

**Published:** 1888-03

**Authors:** 


CONSTITUTION AND BY-LAWS.—[In response to frequent requests for copies of the constitution and by-laws, and having none on hand, we publish them here for the information of members and others.]

### CONSTITUTION OF THE P. M. B. A. OF TEXAS.

### ARTICLE I.

Sec. 1. This association shall be known as "The Physicians' Mutual Benefit Association of Texas."

Sec. 2. The office of said association shall be in Fort Worth, Texas. [Removed to Austin by vote of Directors.]

Sec. 3. The object of this association shall be to provide a benefit fund for the beneficiary or beneficiaries of each member at his death.

Sec. 4. The officers of this association shall consist of a President, two Vice-Presidents, Secretary and Treasurer, [the two were blended by vote of the Board of Directors] to be elected by the Board of Directors, and who shall hold their offices till their successors are elected and qualified.

Sec. 5. There shall be a Board of Directors, consisting of seven members, each of whom shall hold his office till his successor is elected and qualified.

ARTICLE II.

Sec. 1. Applications for membership shall be made to the officers of the association, and shall be made upon the printed form furnished by the same, and shall be accompanied by a fee of one dollar ($1), membership fee, the same to be paid annually on the 1st day of January of each year. Upon receipt of application and membership fee, the Secretary shall issue to applicant a certificate of membership, signed by the President and Secretary.

ARTICLE III.

Sec. 1. On the death of a member in good standing the Secretary shall forward to the family of the deceased, the proper blanks for making out proof of death, which, when duly filed with the Secretary, the Board of Directors shall order the Secretary to levy an assessment of one dollar ($1) on each member, and when the same shall have been paid into the treasury, the Board of Directors shall order the Secretary to draw a draft on the Treasurer for the total amount so collected or paid in, [less cost of collecting] said draft being made payable to the person or persons named in the applicants application, as his beneficiary or beneficieries, and the amount thus paid, shall in no case exceed the sum of one thousand dollars.

Sec. 2. Assessments shall be levied on members only upon the death of a member, and for no other purpose than the payment of a death benefit.

Sec. 3. Notices of assessments shall be forwarded to the nearest known post office of the members, which shall be deemed sufficient notification, and if said assessment is not paid within thirty days, the defaulting members shall be suspended from the benefits of the association, and shall forfeit all money previously paid in.

BY-LAWS OF THE P. M. B. A. OF TEXAS.

ARTICLE I.

Sec. 1. The President shall preside at all meetings of the assocition but shall only be allowed a vote in case of a tie. He shall sign all certificates of membership and official papers, and shall have supervision of the books and accounts of the association, and see that all rules and regulations of the association are fully executed.

Sec. 2. The Vice-Presidents shall possess the power and perform the duties of the President in his absence.

Sec. 3. The Secretary shall keep a record of all meetings of the association, do all its correspondence, keep a correct list of its members, and keep an account with each, make all assessments and collect money due the association and pay over the same to the Treasurer, taking his receipt for the same. He shall receive and file all applications and make out and issue all certificates, and for these services he shall receive three hundred dollars ($300) per annum, to be paid quarterly, out of the fund created by the membership fees. For this sum the President shall order him to draw a draft on the Treasurer, and shall countersign the same. The Secretary shall give such bond as the Board of Directors shall direct.

Sec. 4. The Treasurer shall receive all monies due the association from the Secretary, giving his receipt for the same, shall hold the same in safe-keeping and pay the same out only on a warrant by the Secretary, countersigned by the President and Secretary. He shall give such bond as the Board of the Directors may require for the faithful performance of these duties.

Sec. 5. The membership fees shall constitute an expense fund, out of which all expenses of the association as pertain to its workings, and the salary of the secretary shall be paid, and shall be used for no other purpose.

These By-Laws, or any of them, may be amended or suspended by a two-third vote of the Board of Directors, after the proposed alteration or amendment has been submitted to the Board of Directors at least one month before it is adopted.

---

AMERICAN MEDICAL ASSOCIASION.—The thirty-ninth Annual Session will be held in Cincinnati, Ohio, on Tuesday, Wednesday, Thursday and Friday, May, 8, 9, 10 and 11, commencing on Tuesday, at 11 A. M.

---

ANOTHER PRECINCT HEARD FROM.—The Val Verde County, Medical Society, has been organized, and holds its meeting at Del Rio. Dr. E. G. Nicholson was elected president, and Dr. J. R. Harmer, secretary.

